# Object localization using a biosonar beam: how opening your mouth improves localization

**DOI:** 10.1098/rsos.150225

**Published:** 2015-08-26

**Authors:** G. Arditi, A. J. Weiss, Y. Yovel

**Affiliations:** 1 School of Electrical Engineering, Faculty of Engineering, Tel Aviv University, Tel Aviv, Israel; 2 Department of Zoology, Tel Aviv University, Tel Aviv, Israel; 3 Sagol School of Neuroscience, Tel Aviv University, Tel Aviv, Israel

**Keywords:** biosonar, bats, sensory systems, beam, neuroscience, model

## Abstract

Determining the location of a sound source is crucial for survival. Both predators and prey usually produce sound while moving, revealing valuable information about their presence and location. Animals have thus evolved morphological and neural adaptations allowing precise sound localization. Mammals rely on the temporal and amplitude differences between the sound signals arriving at their two ears, as well as on the spectral cues available in the signal arriving at a single ear to localize a sound source. Most mammals rely on passive hearing and are thus limited by the acoustic characteristics of the emitted sound. Echolocating bats emit sound to perceive their environment. They can, therefore, affect the frequency spectrum of the echoes they must localize. The biosonar sound beam of a bat is directional, spreading different frequencies into different directions. Here, we analyse mathematically the spatial information that is provided by the beam and could be used to improve sound localization. We hypothesize how bats could improve sound localization by altering their echolocation signal design or by increasing their mouth gape (the size of the sound emitter) as they, indeed, do in nature. Finally, we also reveal a trade-off according to which increasing the echolocation signal's frequency improves the accuracy of sound localization but might result in undesired large localization errors under low signal-to-noise ratio conditions.

## 1. Introduction

Accurately determining the location of a sound-emitting source can have a huge influence on an animal's fitness. Because movement usually produces sound, animals (humans included) have developed neural and morphological mechanisms to enable precise sound localization [1–4]. Localization performance could significantly affect survival such as in the case of localizing the rustling sound of a sneaking predator or a moving prey. It is well known that animals use binaural and monaural temporal and spectral cues in order to localize a sound source (i.e. its azimuth and elevation [1–4]). Echolocating bats emit sound in order to sense their surroundings. Like other mammals, bats have been shown to rely on binaural and monaural temporal and spectral cues when localizing a sound-emitting organism or a sound-reflecting object [5–11]. The active nature of their sensory system, however, provides bats with additional spatial information which is encoded in their sonar beam.

**Figure 1.**
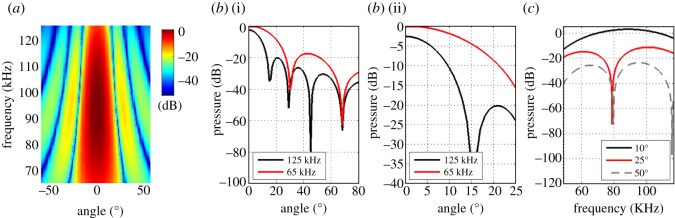
Spatial information in the emitted beam. (*a*) Beam directionality for different angles and frequencies according to the piston model. (*b*) A cross section through the full beam description (*a*) is shown for two frequencies (65 and 125 kHz) with the sector between 0 and 25 degrees enlarged on the right (*b*(ii)). Note how an object located at 12 degrees will reflect *ca* 13 dB less pressure at 125 kHz relative to 65 kHz. We show how this difference can be used to infer the angle of the object. (*c*) The spectrum of an echo reflected from an object positioned at three azimuths (10, 25 and 50 degrees) will be different due to the frequency-dependent directionality of the emitted beam. Data in this figure were generated using a 6.3 mm radius piston representing *Myotis emarginatus* (table 2). The spectrum of this signal was generated using a Kaiser window (see Methods) and is typical for vespertilionid bats in general and *M. emarginatus* specifically.

Because of the physics of sound propagation, the beam emitted by a bat is directional in space [12–17]. In bats that use a range of frequencies (e.g. bats using frequency-modulated—FM—signals), the directionality of the beam at each frequency varies greatly, with higher frequencies spreading into a narrower sector of the environment (figure 1*a*,*b*). This means that an object positioned at different angular locations (azimuth or elevation) relative to the bat will be impinged on by a different spectrum of frequencies and will therefore reflect a different spectrum (figure 1*c*). The spectrum of the echo can therefore provide spatial information about the position of the object from which it was reflected. Indeed, there is some evidence that active (echolocation-based) sound localization in bats is more accurate than passive sound localization (*ca* 15 versus *ca* 1.5 degrees; compare [6] and [10]).

Most previous studies on sound localization in bats ignored the information available in the emitted beam and focused on cues allowing passive sound localization [7,9,11,18–20]. Passive sound localization cues are typically used by mammals and are generated from the filtering of the received sound by the ears and the head of the animal. Apart from this head-related transfer function (HRTF), passive localization cues also include interaural time and intensity differences.

Several previous studies used a comprehensive model which included both the spatial filtering of the bat ears and the emitted beam directionality [21–24]. However, they did not assess the specific contribution of the emitted beam alone to sound localization. Moreover, they did not examine how the spatial information provided by the beam varies as a function of the echolocation signal and as a function of the morphology of the sound emitter (i.e. the mouth gape).

Here, we concentrate on the spatial information provided by the bat's echolocation beam only and on the control that bats might have over the acquisition of this information. We will deal with only angular localization and not with estimating the range of an object that can be very accurately calculated from the echo–pulse time delay [25,26]. We therefore refer to the azimuth and elevation angles of an object as its position.

As explained previously, the location of an object can be determined based on its echo's spectrum which results from the angle-specific spectrum of the emitted beam. In theory, if the bat were able to emit a beam with a *unique* frequency spectrum towards each spatial angle (azimuth and elevation combination), such a beam would encode the environment exclusively which would allow sound localization with no reliance on additional cues (i.e. the bat would not need external ears and would manage with one ear). However, owing to the emission properties of sound, two objects at nearby angles will always be impinged on by correlated spectra thus limiting spatial resolution when using the emitted beam. We restricted our analysis to orally emitting bats (the big majority of echolocating species) whose sound beam can be approximated using the piston model [15,27]. We examined the information conveyed by the emitted beam only, and made no assumptions regarding the spatial information provided by the ears (HRTF) which was dealt with elsewhere [7,11]. Our results thus present a lower bound on localization performance which could be further improved if the HRTF is used. The main aim of our work was not to estimate absolute localization performance as this depends on many more parameters (e.g. the HRTF) but to assess how different factors (e.g. the echolocation signal design) affect beam-based localization.

We found that bats' biosonar beams provide vast spatial information about the angular position (azimuth and elevation) of a reflecting object that could be used by the bat for angular localization. We show how bats could significantly improve the performance of angular localization by adjusting their beams via alteration of the echolocation signal design or the emitter size (i.e. their mouth gape). We describe a new trade-off which makes different signal designs advantageous for spatial localization under different noise conditions. Namely, we show that using higher signal frequencies improves the localization accuracy, and could also increase the localization error under low signal-to-noise ratio (SNR) conditions.

## 2. Results

In order to use the emitted beam to localize a reflecting object, a bat needs to estimate the angle of an object given the spectrum of its reflected echo and given the emitted beam. Intuitively, this should be done by comparing the spectrum of the received echo with the spectrum that was transmitted into each angle, and finding the most similar pair. We derived the maximum-likelihood estimate for this problem. This derivation suggests that the best way to estimate the angle is the template matching solution which correlates the (amplitude) spectrum of the received echo with the expected spectra at all angles; and takes the angle that maximizes this measure (after normalization, equation (2.1) and see Methods in the electronic supplementary material for the full derivation):

2.1 $$\hat{\theta }=\arg\max\frac{{\left|\text{g}{\left(\theta \right)}^{T}\text{y}\right|}^{2}}{{\left|{\text{g}}^{T}(\theta )\text{g}(\theta )\right|}^{2}},$$

where $\hat{\theta }$ is the estimated angle of the reflecting object, g(*θ*) is the expected amplitude spectrum for each angle *θ* (which was estimated using the piston model, see Methods) and *y* is the amplitude spectrum of the received echo. ()^T^ denotes the transpose operation.

**Figure 2.**
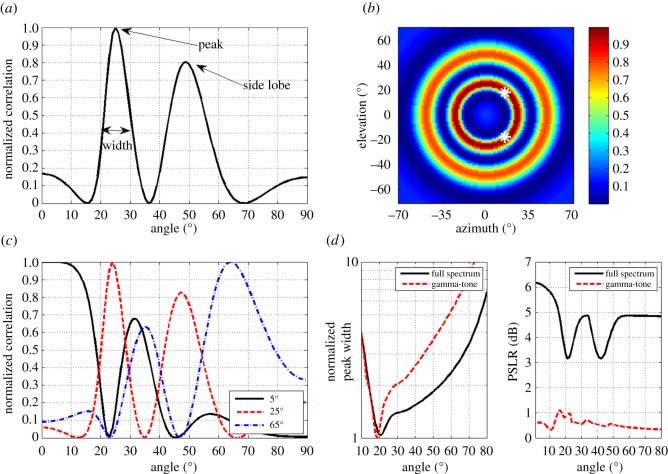
Angle estimation using the emitted beam. All panels were calculated using an FM signal of 130–40 kHz and a 6.3 mm radius aperture mimicking the signal and mouth gape of *M. emarginatus*. (*a*) The correlation function for an object at 25 degrees. The correlation of the reflected echo spectrum with the expected spectra of different angles yields a maximum at the right angle—25 degrees. Note that there is a side lobe (a potential error) at *ca* 50 degrees. (*b*) Two-dimensional correlation map between the spectrum of the echo and the beam's spectrum. Hot colours depict high correlation. Note that the solution is circular symmetric—assuming that the range was estimated by the bat based on the pulse–echo delay. If for instance we assume that the azimuth of the object was determined via ITD, as 20 degrees, then only two symmetric solutions (above and below the horizon) are possible (white asterisks). (*c*) The same as in (*a*) but for three different objects located at angles 5, 25 and 65 degrees. Note how the main lobe at different angles varies in width and how side lobes appear for certain angles. (*d*) Left: angular accuracy—the width of the main lobe of the correlation function (see *a*) for different angles when using the full spectrum (black) or the gamma-tone filter (red). Right: angular ambiguity—the peak to side-lobe ratio for different angles when using the full spectrum (black) or the gamma-tone filter (red).

We started off examining the spatial information provided by the wideband *Myotis*-like signal (mimicking a *Myotis emarginatus* signal). We simulated the beam of a linear FM down sweep ranging between 130 and 40 kHz [28] with an appropriate *M. emarginatus* mouth gape (6.3 mm, estimated based on fig. 4 in [13]). We made no assumptions about the bat's HRTF or about the object's distance and frequency response (see Methods). This analysis revealed that the bat's echolocation beam alone provides vast spatial information about the position of an object. We estimated the localization performance using the angular correlation function. This function summarizes the correlation between the actual received spectrum of an echo and the expected spectrum for all angles (figure 2*a*). The peak of the angular correlation function depicts the angle that is most likely to be the angle of the object. Assuming that the range of the object was estimated using the pulse–echo time delay, and assuming a symmetric beam (as suggested by the piston model and by data [27]), there will always be circular ambiguity, when estimating the position of an object based on the spectral information conveyed by the emitted beam (see the ring of maximum correlation in figure 2*b*). Under natural conditions, this ambiguity could be solved by using additional cues such as the temporal or spectral information available in the HRTF. For instance, if the bat estimates azimuth based on interaural level or time differences (ILD or ITD, as is often assumed for mammals), then the circular ambiguity converges to only two possible elevations (the intersection of two circles, see white asterisks in figure 2*b*). These two possible solutions could then be distinguished between based on additional monaural spectral cues. Hence, beam-based spatial information should be thought of as additional spatial information (additional to that provided by the HRTF and the ILD/ITD). Alternatively, a moving bat could analyse two consecutive echoes from different angles and estimate the intersection of two circles resulting in two possible positions. The bat could even use three consecutive echoes to eliminate this dual ambiguity and remain with a single point. This means that, in theory, position could be estimated based on the spatial information conveyed by the emitted beam only.

It should be noted that the correlation function often contained side lobes (figure 2*a*–*c*) which could be mistaken for the main lobe in low SNR conditions. These side lobes arise when the spectra transmitted into two angles are similar such as the spectra at 25 and 50 degrees (figure 1*c*) which results in the side lobe at 50 degrees in figure 2*a*. We will discuss the effect of these side lobes below.

**Figure 3.**
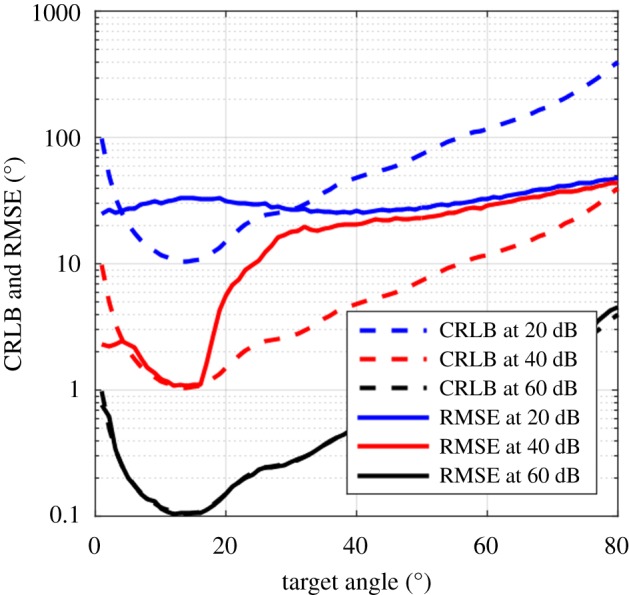
Localization performance enabled by the emitted beam only. The Cramer–Rao lower bound (CRLB) and the RMSE of absolute theoretical angular accuracy are presented when using the biosonar beam only. The CRLB and RMSE were estimated for three SNRs—20, 40 and 60 dB. Note that the CRLB and RMSE at 60 dB fully overlap and thus the black dashed line is not visible.

It is difficult to estimate the absolute localization accuracy in degrees provided by the emitted beam. In order to do so, one must know the external noise and the internal noise in the bat's receiver (i.e. its detection threshold) and take species-specific factors into account such as the HRTF. We thus mainly focus on a comparative analysis of the information provided by different signals and not on the absolute available information. However, to get a rough estimation of the localization performance enabled by the beam-only, we used two analyses. (1) We used the Cramer–Rao lower bound (CRLB) in order to evaluate the absolute theoretical angular accuracy when using the biosonar beam only under different noise levels (20–60 dB). This lower bound reflects the best localization accuracy that can be achieved for a given SNR (equivalent to the ‘just noticeable difference’ often used in psychophysics; see electronic supplementary material, Methods for its derivation). (2) We ran Monte Carlo simulations, simulating beams under different noise levels (20–60 dB), and ran the maximum-likelihood analysis to estimate the mean error at different angles. Unlike the CRLB, this estimate takes side-lobe ambiguities (see above) into account. When assuming an SNR of 40 dB which is reasonable for a loud echo in a quiet environment [29,30], our analysis suggested that localization based on the information conveyed by the beam of *M. emarginatus* can be as accurate as 1 degree. When assuming an SNR of 60 dB, localization accuracy improved by an order of magnitude and the opposite happened for a 20 dB SNR (figure 3). At high SNR levels, the Monte Carlo simulations predicted similar performance. However, at low SNR, the performance decreased owing to side-lobe ambiguities, mainly at angles larger than 20 degrees (figure 3).

Best localization was achieved around 15 degrees (relative to the centre of the emitter). Better localization is achieved when the spectrum of a specific angle is unique. Spectral notches, for example, can contribute to uniqueness (as is the case at 15 degrees; figure 1*b*(i)). More generally, better localization is reached when the spectrum changes fast across angles, so high accuracy will be achieved where the piston–beam has a high slope.

These localization estimations can be improved by the bat by altering the echolocation signal design or the mouth gape. In the following sections, when aiming to compare the effects of different factors (e.g. the signal design) on the localization performance, we used two measures (figure 2*a* and see Methods). (1) The width of the main lobe of the angular correlation function. This is a measure of angular accuracy with a narrow width predicting more accurate localization. (2) The peak-to-side lobe ratio (PSLR) which was defined as the ratio between the height of the peak of the correlation function and the height of its main side lobe. High PSLR values are advantageous because they reduce the probability that the side lobe will be mistaken for the main lobe. This measure is thus an estimate of how localization is robust to noise or how ambiguous is localization in a noisy environment. We will refer to these two parameters as the angular accuracy and the angular ambiguity of localization.

Angular accuracy and angular ambiguity are presented for an FM signal between 130 and 40 kHz mimicking the signal of *M. emarginatus* (figure 2*d*, black lines).

As explained above, the actual performance of a bat depends on the exact noise in its system and has to be tested behaviourally. Therefore, the values in all graphs in this article are normalized and should not be taken as absolute localization estimates as these depend on the SNR. The results should be interpreted in a relative fashion to compare the influence of different factors (e.g. the signal design) on localization. We normalized each plot by the minimum of all curves and thus the minimum is always 1 degree. The relative values are meaningful: an increase from a width of 1 to 3 denotes a threefold increase in width or a threefold decrease in accuracy.

Until now, we used the raw amplitude spectrum of the signal in our analyses. A more biologically plausible approach would be to use the gamma-tone filter-bank which is commonly accepted as a good model of the mammalian inner ear filtering [31,32] (see Methods). Overall, the gamma-tone model behaved similarly to the full spectrum model. It provided similar angular accuracy at small angles and was slightly inferior at large angles (compare red and black lines in figure 2*d*). Its PSLR was 2–5 dB smaller across all angles (suggesting more ambiguity) but the general pattern was similar (i.e. the ratio jittered around an average value for all angles). We ran all analyses below with both the gamma-tone and full spectrum models and the trends were similar, i.e. factors that improved/reduced localization in one model also did so in the other one. As this work is a theoretical analysis of available information, we show the results for the full spectrum analysis in the remainder of the paper.

**Figure 4.**
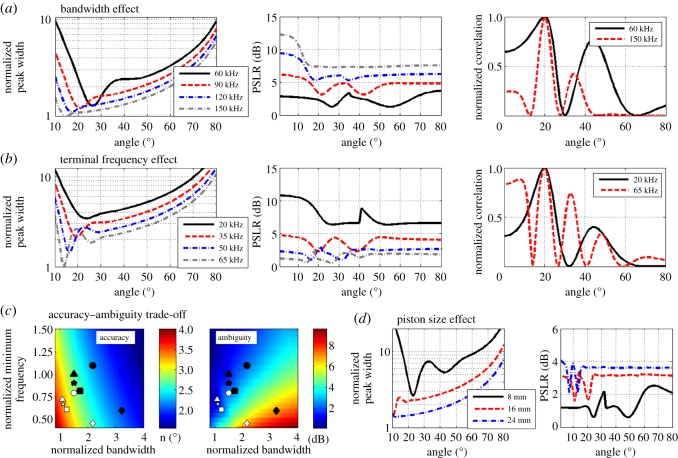
The effect of signal design on localization. In all panels, the mouth gape was fixed at 8 mm. (*a*) Localization improves with bandwidth. Both angular accuracy (left) and peak-to-side lobe ratio (middle) improve as a function of bandwidth (the terminal frequency was fixed at 30 kHz). Right—the correlation function for two bandwidths shows how the main lobe narrows and the side lobes decease with bandwidth. (*b*) Decreasing the terminal frequency improves the angular accuracy but decreases the peak-to-side lobe ratio thus increasing the angular ambiguity in low SNR. Here, the bandwidth was held constant (90 kHz). Left, middle, right—like in panel (*a*). Note how in the correlation function (right) a higher terminal frequency (red) narrows the main lobe, improving angular accuracy, but also increases the height of the side lobes thus increasing angular ambiguity. (*c*) The accuracy–ambiguity trade-off. The effect of the bandwidth and terminal frequency on accuracy (left) and ambiguity (right). Both accuracy and ambiguity are colour-coded, but note that for accuracy (left) small values (blue) are beneficial while for ambiguity (right) high values (red) are beneficial. The *y*-axis shows the normalized terminal frequency, *a*/λ_1_, and the *x*-axis shows the normalized bandwidth, *a*/λ_1_−*a*/λ_2_, where *a* is the mouth gape radius, λ_1_ the terminal-frequency wavelength and λ_2_ the start-frequency wavelength. The symbols represent the values used by five vespertilionid bats with their mouth half open (white) and fully open (black). Note how black symbols always provide better accuracy and less ambiguity. Triangles, *M. dasycneme*; diamonds, *M. nattereri*; squares, *M. mystacinus*; circles, *M. emarginatus;* asterisks, *M. daubentonii.* (*d*) Increasing the mouth gape improves both angular acuity and angular ambiguity. The signal remained constant during simulation (30–110 kHz) while only the piston size was altered.

We next tested the effect of the bat's signal design on the angular localization facilitated by the emitted beam. We simulated different FM sweeps and tested the effect of the signal's bandwidth and its terminal (lowest) frequency, two parameters that greatly vary between bat species. All signals that were compared in this study always had the same total power (see Methods). We found that increasing the bandwidth improved localization in terms of both the angular accuracy and the angular ambiguity (figure 3*a* and electronic supplementary material, figure S1). The terminal frequency on the other hand had an opposite effect on the two aspects of localization. Higher terminal frequencies (under constant bandwidth) improved the accuracy of angular localization. However, they also increased the angular ambiguity (by decreasing the PSLR, figure 4*b*; and electronic supplementary material, figure S1) which could be disadvantageous in low SNR situations. These results reveal a trade-off between angular accuracy and angular ambiguity which are affected by the signal design in opposite directions (figure 4*c*).

We also compared uni- and multi-harmonic signals to examine how weighting the spectrum differently affects localization. The terminal frequency, the bandwidth, and the total power of the two signals were the same but the multi harmonic signal had five harmonics (simulating an approach call of a *Rhinopoma microphyllum* bat [29]). The overall performance of the multi harmonic signal was similar to that of the uniharmonic signal. The multiharmonic signal performed slightly worse in terms of angular accuracy, but it was better in terms of angular ambiguity generating higher PSLRs (electronic supplementary material, figure S2).

A bat could also change its beam by altering the aperture of the piston, i.e. by altering its mouth gape. We found that by opening its mouth, the bat can overcome the accuracy–ambiguity trade-off and improve angular acuity without impairing angular ambiguity (figure 4*d*; and electronic supplementary material, figure S1). The improved performance results from an increase in the effective bandwidth (see Discussion). Figure 4*c* and table 1 summarize the effect of signal bandwidth and terminal frequency on beam-based localization performance. The white symbols in the figure represent the spatial information provided by the signals of five vespertilionid bats whose localization performance has been studied [28] (and see table 2). Among these bats, *M. nattereri* stands out with a signal that is expected to provide better performance as was indeed the case in the behavioural experiments [28]. By opening their mouth, all five bat species could further improve spatial localization (compare white and black symbols) while paying a cost in narrowing the sector of space that they are scanning. On average, accuracy improved by 10 degrees and ambiguity by 0.3 dB (table 1). Interestingly, we have recently shown that echolocating bats widen their mouth gape when entering confined—highly cluttered—spaces [33]. This behaviour narrows the overall beam thus decreasing background echoes. However, as we show here, increasing the mouth gape would also improve beam-based spatial localization.

**Table 1.** Opening the mouth gape improved both localization accuracy (expressed by the narrowing of the width of the correlation function peak) and localization ambiguity (expressed by the increase in the peak to side lobe ratio of the correlation function).

	accuracy—peak width (degree)		ambiguity—PSLR (dB)	
species	mouth closed	mouth open	mouth closed	mouth open
*M. nattereri*	13.9	7.6	6.8	7.0
*M. emarginatus*	17.2	9.2	2.4	2.4
*M. mystacinus*	25.6	14.3	1.9	2.6
*M. daubentonii*	28.1	15.7	1.4	1.7
*M. dasycneme*	27.1	15.0	1.1	1.5

**Table 2.** Signal and mouth parameters of the five vespertilionid bat species that were examined. Bandwidth was determined according to a drop of 25 dB relative to the peak. Data were taken from [12,28].

species	piston radius (mm)	terminal frequency (kHz)	bandwidth (kHz)
*M. nattereri*	7.6	19	119
*M. emarginatus*	6.3	42	91
*M. mystacinus*	5.4	32	75
*M. daubentonii*	6.1	33	57
*M. dasycneme*	8	30	44

## 3. Discussion

Our results demonstrate the great potential of using the spatial information generated by the biosonar beam for localization. Behavioural results have indeed suggested that the angular localization performance in bats is better when they rely on active biosonar than when they passively listen to sounds (compare [6] and [10]). We used the piston model to simulate the biosonar beam, but our results should generally hold for any emitter which can be characterized by frequency-dependent directionality. This is most likely the case for the great majority of echolocating bats. Our analysis should also be relevant for echolocating toothed whales, especially those with wideband signals [34].

We show how the biosonar signal design affects the spatial information available in the beam. A recent study has suggested that bats' biosonar beams have been adapted throughout evolution to contribute to spatial localization [13]. Here, we suggest how the exact signal design (i.e. its spectral content) could have evolved to facilitate localization. In the past, the biosonar signal design was mostly studied and discussed in terms of ranging accuracy and Doppler tolerance [28,31,35,36]. Without diminishing the importance of signal design in ranging, we suggest that localization performance could have also played a role in shaping the spectrum of extant signal designs used by bats today.

We found that increasing the signal's bandwidth improves angular localization. This is perhaps intuitive owing to the additional information gained when using a wider range of frequencies which reduces the similarity of spectra directed in different angles. As wider bandwidth signals are advantageous for both ranging accuracy [36] and localization performance, evolution could increase signal bandwidth to improve both. However, sensory adaptations usually come at a cost. The disadvantage of a wide bandwidth signal in terms of detection range has been discussed elsewhere [37]. Echolocating bats typically increase their bandwidth when approaching an object or a prey item [37]. This increase can sometimes reach a few dozens of kilohertz [38] and is usually attributed to the need to improve ranging performance when approaching [35,37]. Once again, we argue that this could also facilitate better angular localization which is needed when closing in on a target.

The effect of the signal's terminal frequency on beam-based localization is less straightforward. On the one hand, higher terminal frequencies improve angular accuracy by narrowing the main lobe of the angular correlation function. On the other hand, they also increase the height of the side lobes of this function. This means that when the bat is operating at high SNR (e.g. in quiet environments, or when localizing large objects) high terminal frequencies are advantageous. But in a noisy environment, high side lobes pose a danger, because they can be confused for the main lobe which will result in large errors. This accuracy–ambiguity trade-off might therefore be relevant for bats that are active under different SNR conditions. Our results therefore suggest that to improve beam-based localization, evolution should have driven higher terminal frequencies, but that this should have been accompanied by a parallel increase in bandwidth to avoid ambiguities.

Clearly, more considerations other than ranging and beam-based localization might have played a role in the evolution of signal design. Among those are detection abilities and other localization considerations such as the HRTF (i.e. a signal could have evolved to provide more ear-related spectral information). It should be emphasized that the beam-induced spectral cues cannot be accessed by the bat's brain independently of the effect of the HRTF. The spectrum of the reflected echo is a result of both beam directionality and the HRTF so these two factors (and the object's frequency response) will influence localization. Here, we focused on the less studied factor—the emitted beam without assuming anything about the other two: the HRTF and the object's frequency response. We thus maintained these two factors constant and varied the one under examination. Our results thus provide a general framework and are not specific to any bat species or target. Importantly, the basic principles we found (e.g. how bandwidth, terminal frequency and mouth gape influence beam-based information) would not change and are independent of the chosen HRTF. As the HRTF is a complex mixture of troughs and peaks (at different azimuth–elevation combinations), it could be thought of as additional spectral information which is ‘riding’ on top of the angle-specific spectrum resulting from beam directionality.

Aside from changing their signal, we demonstrate how bats could improve localization by widening their mouth gape. This simple strategy results in better angular accuracy and slightly less angular ambiguity. Evolution could therefore push an ability to increase mouth gape as an alternative to changing signal design. Naturally, opening the mouth comes at a cost—a decrease in the total sector scanned by the bat equivalent to a decrease in its biosonar ‘field of view’ (increasing the terminal frequency would have a similar effect). This cost might be the reason for the finding of Jakobsen *et al.* [13] that bats do not operate with their mouth gape open at its maximum span.

The reason for the improved performance achieved by opening the mouth is the increase in the beam's effective bandwidth (*a*/λ_2_−*a*/λ_1_, when *a* is the emitter diameter, λ_2_ is the wavelength of the highest frequency and λ_1_ of the terminal frequency of the signal). Shorter terminal wavelengths (λ_1_) representing higher terminal frequencies increase accuracy but they also induce higher side lobes. Opening the mouth (increasing *a*) will have the same effect as decreasing λ_1_ on accuracy; however, by increasing *a* the effective bandwidth also increases and thus side lobes are attenuated. For example, if a bat is using a 30–60 kHz signal (equivalent to wavelengths of *ca* λ_1_=1 cm to *ca* λ_2_=0.5 cm), when its mouth is open at a radius of 1 cm, the effective bandwidth of its beam is 1 (*a*/λ_2_−*a*/λ_1_=2−1). Increasing the mouth gape to 2 cm would then increase the effective terminal frequency (*a*/λ_1_) but it will also increase the effective bandwidth which will now reach 2 (*a*/λ_2_−*a*/λ_1_=4−2). Therefore, opening the mouth is equivalent to both increasing terminal frequency and increasing bandwidth. Importantly, we have recently shown that bats increase their mouth gape when entering highly cluttered environments [33]. A simple explanation for increasing the mouth gape in high clutter is narrowing the beam to eliminate background echoes. Our analysis in this work suggests another advantage for increasing the mouth gape in such situations—improving localization. Our results might explain why bats sometimes open their mouth widely [33] even though this behaviour seems not to be related to emission intensity [39].

To test our framework for beam-based localization on extant echolocating bats, we examined five species of the vespertilionid family for whom the mouth gape was estimated [13,27] and their performance in detecting insects in front of background clutter (an artificial clutter screen) was documented [28]. Among these five bats, *M. nattereri* stood out as the species whose beam should enable best localization performance resulting from its ultrawide bandwidth. Interestingly, this bat has also exhibited the best performance in catching prey near background [28]. Its better ability relative to the other bats was explained by the improved ranging performance that its wideband signal should facilitate. The wideband signal of this species will of course contribute to better ranging performance; however, here we show that it should also improve angular localization which could be of high importance in such a task. It would be interesting to estimate the localization performance of bats that can open their mouth very wide, but data on the mouth gape used by echolocating bats are currently missing.

In our analysis, we do not model two effects that could change the spectrum of the received echo thus interfering with the localization approach we describe. We do not model the frequency response of the object which alters the spectrum it reflects and might also be angle-dependent (directional). We also do not treat the fact that atmospheric attenuation is frequency-dependent and thus will also affect the spectrum of the reflected object. One could suggest hypothetical solutions for these problems. For instance, the effect of atmospheric attenuation could be compensated for once the bat has estimated range, and the frequency response of many small insects is relatively flat or might be known to the bat from experience. However, it should be emphasized that these difficulties also exist in passive sound localization where the spectrum of the source and its range are unknown. Our work aimed to probe how much information is available in the emitted beam only and how a bat could influence it. We shed new light on both issues.

## 4. Methods

All simulations were performed in Matlab. We did not model the bat's HRTF, the object's frequency response and the signal's atmospheric attenuation. These effects can be added in order to model a specific bat species or a specific target. Our model provides a general framework for the emitted beam only. The maximum-likelihood analysis (see below) does not include noise. This analysis was therefore used to compare the localization performance enabled by different echolocation signals and different mouth gapes, but not to estimate absolute performance. The CRLB and Monte Carlo analyses (see below) were performed under different noise levels and were used to estimate absolute localization performance.

### 4.1 Simulating the signals and the echoes

The transmitted signal at a given *θ* (azimuth or elevation), *G*_*T*_(*θ*,*t*), was an FM chirp, multiplied by the corresponding envelopes

4.1 $$G_{T}(\theta ,t)=\sum_{p=1}^{M}x_{p}(t)A_{p}(t)h(\theta ,f_{p}(t)),$$

where *h*_*p*_(*θ*,*f*_*p*_(*t*)) is the angle-dependent envelope (spectrum) determined by the piston model (see below), *f*_*p*_(*t*) is the instantaneous frequency of the FM chirp and *A*_*p*_(*t*) is the envelop window which was simulated as a Kaiser window with a constant *β*=4. *p* is the harmonic index (of the *M* harmonics). *p* and *M* were set to 1 in all cases except for the multiharmonic signal. *x*_*p*_(*t*), the instantaneous pressure, is given according to the typical linear sweep equation

4.2 $$x_{p}(t)=\cos\left(2\pi f_{0,p}t+\frac{1}{2}m_{p}t^{2}\right);\;p=1\ldots M,$$

where *f*_0,*p*_ is the starting frequency of harmonic *p* and *m*_*p*_ is the frequency decrease rate per harmonic.

In most cases, a single harmonic chirp was simulated and *M* was thus set to 1, whereas *m*_1_ was determined according to the simulated bandwidth and duration. The spectrum of the simulated signals was convex peaking between the high and terminal frequencies as is the case for many vespertilionid signals. All signals were 3 ms long. For the multiharmonic signal, we simulated a five harmonic signal recorded from *Rhinopoma microphyllum*. The first harmonic (which is almost silent) of the signal was at 12–16 kHz, so the bandwidth of the five simulated harmonics was 24–96 kHz. All signals that were compared always had the same total power.

### 4.2 The piston model

Beam directionality was estimated using the piston model:

4.3 $$h(\theta ,f(t))=\frac{\mathrm{abs}[2J_{1}((2\pi f(t)/c)\cdot a\cdot \sin\theta )]}{(2\pi f(t)/c)\cdot a\cdot \sin\theta },$$

where *h*(*θ*,*f*(*t*)) is the ratio between pressure on-axis and the pressure at an angle *θ* at an instantaneous frequency *f*(*t*). *J*_1_ is the first order Bessel function of the first kind, *a* is the radius of the piston (or the mouth gape) and *c* is the sound propagation speed set to 343 m s^−1^.

### 4.3 The correlation function and its analysis

The correlation function (equation (2.1)) was used to analyse localization performance of a specific signal. The PSLR (ambiguity) was evaluated as the quotient of the two. The width of the main lobe (accuracy) was estimated using the second derivative of the correlation function at the peak. This strategy was used because the main lobe sometimes did not drop on one side (see the 5 degrees curve in figure 2*c* for example). The peak width was estimated according to

4.4 $$\mathrm{PW}(\theta )={\left[\frac{\partial^{2}}{\partial \theta^{2}}(\bar{L}(\theta ))\right]}^{-1/2},$$

where $\bar{L}(\theta )$ is the correlation function. The estimated peak width is the value of PW(*θ*) at the location of the peak (the correct angle).

The maximum-likelihood analysis (resulting in the correlation function) did not take noise into account. The accuracy values predicted in this study therefore do not reflect performance in absolute angles. In each plot, the curves were normalized according to the lowest value (of all plots). Therefore, *y*-values always start at ‘1’ and denote changes in fold: an increase from 1 to 3 reflects a threefold decrease in accuracy. In order to estimate the absolute performance, we evaluated the CRLB (see the electronic supplementary material, Methods). Here, noise was added as independent Gaussian noise in each frequency band. We added noise to simulate three noise conditions: 20, 40 and 60 dB SNR. To assess the effect of side-lobe ambiguities, we used Monte Carlo simulations. For different angles, we simulated the (noisy) spectra at three noise levels (20, 40 and 60 dB) and then used the maximum-likelihood to estimate the angle from the noisy spectrum. The RMSE of this analysis is presented in figure 3.

### 4.4 Gamma-tone filter bank processing

The commonly used Gamma-tone filter bank was applied [40]:

4.5 $$\left.\begin{array}{rl} h_{\mathrm{GT}}{\left(t\right)}^{k}= & \underset{\mathrm{envelope}}{\underbrace{\alpha t^{\left(n-1\right)}e^{-2\pi b_{k}t}}}\underset{\mathrm{carrier}}{\underbrace{\cos(2\pi f_{ck}t+\phi )}},\;t>0\\ k= & 1,2,\ldots N_{F}, \end{array}\right\}$$

where *h*_GT_(*t*)^*k*^ is the *k*th filter's impulse response, *k* is the filter index, *N*_F_ is the number of filters, *n* is the filter order, set to 4 to mimic a mammalian cochlea, *α* is the gain constant, *f*_*ck*_ is the centre frequency of filter *k*, *ϕ* is the phase of the impulse response and *b*_*k*_ is the time constant of filter *k*, set to 0.74*f*_*ck*_, making the bandwidth of each filter 13.5% of its centre frequency.

In order to generate a filter bank which is equally spaced along the logarithmic scale, the centre frequencies are calculated according to [31]

$$f_{ck}\;(\mathrm{Hz})=5703\cdot 2^{k/13.5}.$$

## Supplementary Material

Supplementary Figures

## Supplementary Material

SUPPLEMENTARY METHODS

## Data accessibility

All codes and data are available at http://dx.doi.org/10.5281/zenodo.21155.
