# Supplementary material for: Object localization using a biosonar beam: how opening your mouth improves localization
Source: R Soc Open Sci. 2015 Aug 26;2(8):150225. doi: 10.1098/rsos.150225 (PMC4555857; doi:10.1098/rsos.150225)
Supplement: Supplementary Figures [file rsos150225supp1.docx]

**Supplementary Figures**


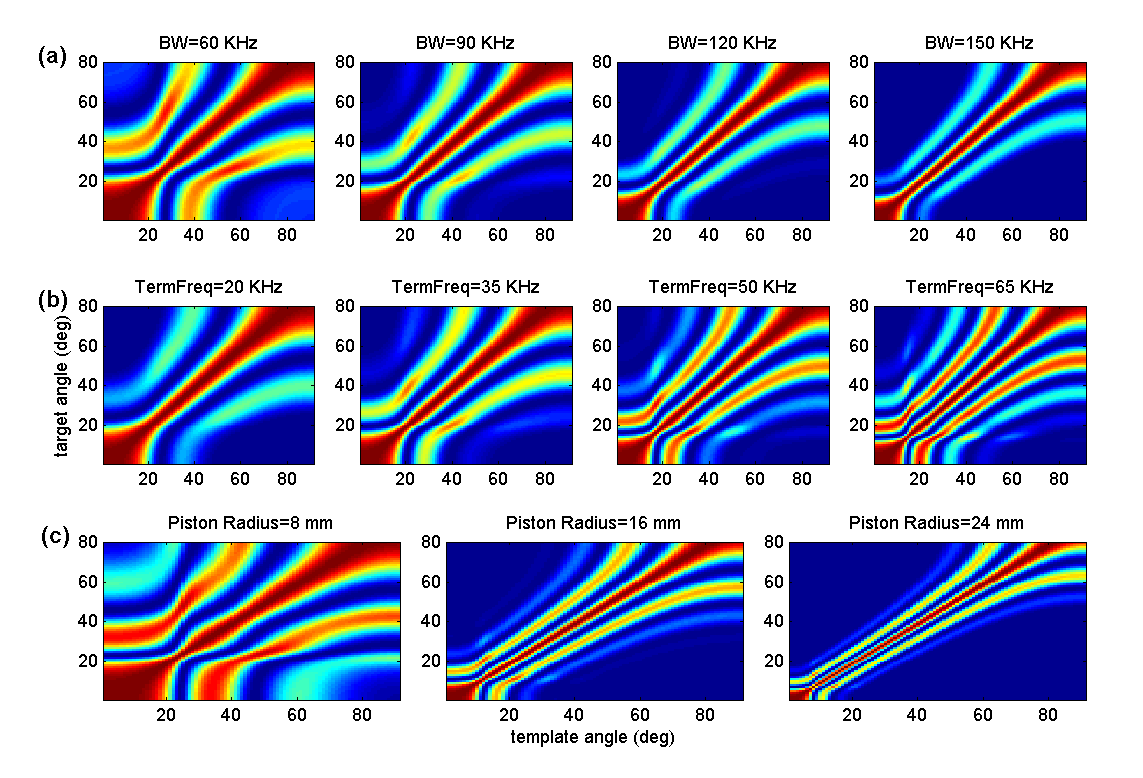


**Supplementary Figure 1**. The correlation surface shows the correlation function for all angles. The x-axis shows the correct angle of the object while the y-axis shows the correlation function. Each column in this matrix is a correlation function for a specific angle (like the ones presented in Figure 2a, 2c). The middle diagonal ridge reflects the main lobe and the additional ridges depict side-lobes. One can thus learn about the influence of different factors on both the width of the main lobe and the height of the side-lobes. Here we show the correlation surfaces for three parameters: (a) first row - bandwidth, (b) second row - terminal frequency and (c) third row - mouth gape.


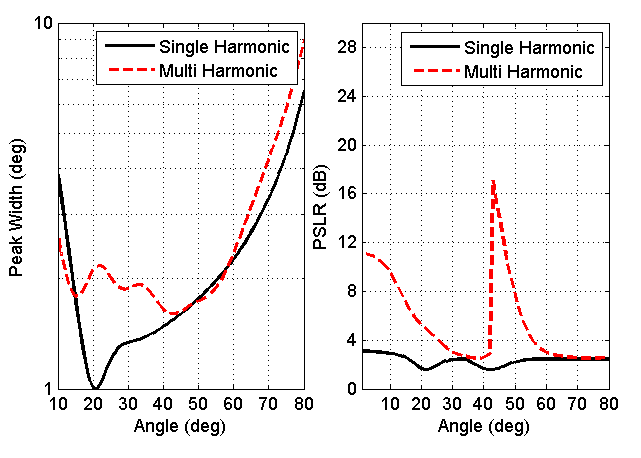


**Supplementary Figure 2.** Comparison of the performance of a uni-harmonic (black) signal and a multi-harmonic (red) signal. Both signals swept between 96-24 kHz and had the same total power.
