# Supplementary material for: Object localization using a biosonar beam: how opening your mouth improves localization
Source: R Soc Open Sci. 2015 Aug 26;2(8):150225. doi: 10.1098/rsos.150225 (PMC4555857; doi:10.1098/rsos.150225)
Supplement: SUPPLEMENTARY METHODS [file rsos150225supp2.pdf]

1. Supplementary Methods

1.1. Signal Model

Each harmonic sweep can be expressed as a linear chirp of 3ms duration, where the instantaneous frequency is:

$$f_p(t) = f_{0,p} + m_p t;\ p = 1..M \qquad (1)$$

Where:

- $f_{0,p}$ - The starting frequency of harmonic 'p'
- m_p - The frequency increase/decrease rate per harmonic.
- M – The number of chirp signals transmitted at the same time

In the above example: m_p=-4*(p+1), $f_{0,p}$=16KHz*(p+1) and M=5.

The transmitted chirp per harmonic 'p' can be expressed as:

$$x_p(t) = \cos\left(2\pi f_{0,p} t + \frac{1}{2} m_p t^2\right);\ p = 1..M. \qquad (2)$$

Next, we consider the fact that the transmitted signal is multiplied by a frequency dependent envelope $A(f)$ known to the bat (which is the transmission transfer function multiplied by the reflector response) and the angle dependent envelope $h(\theta, f)$ which is calculated according to the piston model equation. We can now define the time dependent envelope signals as:

$$\begin{aligned} A_p(t) &\triangleq A\left(f_p(t)\right) \\ h_p(\theta,t) &\triangleq h\left(\theta, f_p(t)\right) \end{aligned} \qquad (3)$$

Therefore, the total transmitted signal at a given θ, $G_T(\theta,t)$, is a superposition of the M chirp signals, multiplied by the corresponding envelopes:

$$G_T(\theta,t) = \sum_{p=1}^{M} x_p(t) A_p(t) h_p(\theta,t) \qquad (4)$$

The total discrete sampled signal can then be written as:

$$g_n(\theta) \triangleq G_T(\theta, nT) = \sum_{p=1}^{M} x_p(nT) A_p(nT) h_p(\theta, nT) = \sum_{p=1}^{M} x_p[n] A_p[n] h_p[\theta, n]; \quad n = 1:N \tag{5}$$

Where:

T - The sampling interval

N – The number of samples (making $(N-1)T$ the total signal duration).

1.2. Maximum Likelihood Estimation

1.2.1. Time Domain M.L. Estimation

For a single reflector located at an angle θ, the discrete signal at the receiver is:

$$y_n \triangleq \alpha g_n(\theta) + v_n; \quad n = 1, 2, ..., N \tag{6}$$

Where α is an unknown attenuation factor depending on distance and target RCS (we assume α to be constant), $g_n(\theta)$ is the transmitted signal according to equation (5) and v_n is the additive noise. Assuming v_n is Gaussian, the pdf of y_n is Gaussian with a mean of $\alpha g_n(\theta)$ and the variance of v_n. Assuming the noise samples are also independent, we can write the joint pdf of **y**, the samples vector, given θ and α as:

$$P_{\mathbf{y}}(\mathbf{y}; \theta, \alpha) = \prod_{n=1}^{N} \frac{1}{\sqrt{2\pi\sigma^2}} e^{-\frac{(y_n - \alpha g_n(\theta))^2}{2\sigma^2}} = \left(2\pi\sigma^2\right)^{-\frac{N}{2}} e^{-\frac{1}{2\sigma^2}\sum_{n=1}^{N}(y_n - \alpha g_n(\theta))^2} = l(\theta, \alpha) \tag{7}$$

Where:

$\mathbf{y} = [y_1, y_2, ..., y_N,]^T$

And $l(\theta,\alpha)$ denotes the likelihood function.

The log likelihood function, $L(\theta.\alpha)$, is then given by:

$$L(\theta, \alpha) = \log(l(\theta, \alpha)) = K - \frac{1}{2\sigma^2} \sum_{n=1}^{N} (y_n - \alpha g_n(\theta))^2 \tag{8}$$

Where K is a constant independent of θ and α.

$L(\theta,\alpha)$ is maximized when the term $\sum_{n=1}^{N}\left(y_n-\alpha g_n(\theta)\right)^2$ is minimized.

Define:

$\mathbf{g}(\theta)=[g_1(\theta), g_2, (\theta)\ldots g_N(\theta)]$

Thus we get

$$\hat{\theta}=\arg\min\left\|\mathbf{y}-\alpha\mathbf{g}(\theta)\right\|^2 \tag{9}$$

The unknown attenuation factor α is estimated (assuming α and θ are uncorrelated) using **g**'s pseudo-inverse matrix, according to:

$$\hat{\alpha}=\left(\mathbf{g}^T\mathbf{g}\right)^{-1}\mathbf{g}^T\mathbf{y} \tag{10}$$

Substituting this term into equation (9) we obtain:

$$\begin{aligned}\hat{\theta}&=\arg\min\left\|\mathbf{y}-\mathbf{g}(\theta)\left(\mathbf{g}^T(\theta)\mathbf{g}(\theta)\right)^{-1}\mathbf{g}(\theta)^T\mathbf{y}\right\|^2= \\ &\arg\min\left[\left\|\mathbf{y}\right\|^2-\frac{\left|\mathbf{g}(\theta)^T\mathbf{y}\right|^2}{\left|\mathbf{g}^T(\theta)\mathbf{g}(\theta)\right|^2}\right]=\end{aligned} \tag{11}$$

$$\hat{\theta}=\arg\max\frac{\left|\mathbf{g}(\theta)^T\mathbf{y}\right|^2}{\left|\mathbf{g}^T(\theta)\mathbf{g}(\theta)\right|^2} \tag{12}$$

It can be seen that the angle is estimated by correlating the received signal vector with the expected signal at each angle and finding the maximum. The denominator contains a normalization term to compensate for the decreasing values of $\mathbf{g}(\theta)$.

1.2.2. Frequency Domain M.L Estimation

For the purpose of calculating the frequency domain M.L estimator, we use the following assumptions:

- Per angle, the available input signal is the magnitude per frequency, available to the bat by gamma-tone filtering.

- The noise is modeled as additive, Gaussian, uncorrelated between different frequencies.

Under these assumptions, it can be seen that equation (6) can be used for the frequency domain model by setting '*n*' as the frequency index and $g_n(\theta)$ as the magnitude per frequency '*n*', per angle θ. The result is that the remainder of the M.L. estimator calculation is identical to that of the time domain case, thus the best angle estimation is obtained by correlating the input frequency domain vector with a set of template vectors and finding the maximum, according to equation (13).

1.3. Cramer-Rao Lower-Bound (CRLB)

Continuing from equation(8), the term for the log-likelihood function can be written as:

$$L(\underline{\theta}) = K - \frac{1}{2\sigma^2}\sum_{n=1}^{N}\left(y_n - \alpha g_n(\theta)\right)^2 \qquad (13)$$

Where:

$\underline{\theta} = \begin{pmatrix} \theta \\ \alpha \end{pmatrix}$ denotes the vector of unknown parameters.

The score is calculated as the derivative of the log-likelihood function:

$$S(\underline{\theta}) = \frac{\partial L(\underline{\theta})}{\underline{\theta}} = \begin{pmatrix} \dfrac{\partial L(\underline{\theta})}{\theta} \\ \dfrac{\partial L(\underline{\theta})}{\alpha} \end{pmatrix} = \begin{pmatrix} \dfrac{\alpha}{\sigma^2}\displaystyle\sum_{n=1}^{N}\left(y_n - \alpha g_n(\theta)\right)\frac{\partial g_n(\theta)}{\partial\theta} \\ \dfrac{1}{\sigma^2}\displaystyle\sum_{n=1}^{N}\left(y_n - \alpha g_n(\theta)\right) g_n(\theta) \end{pmatrix} \qquad (14)$$

And the Fisher Information Matrix (FIM) is:

$$J(\underline{\theta}) = E\left[S(\underline{\theta})S(\underline{\theta})^T\right] = \begin{pmatrix} \left(\dfrac{\partial L}{\partial\theta}\right)^2 & \left(\dfrac{\partial^2 L}{\partial\theta\partial\alpha}\right) \\ \left(\dfrac{\partial^2 L}{\partial\theta\partial\alpha}\right) & \left(\dfrac{\partial L}{\partial\alpha}\right)^2 \end{pmatrix} = \begin{pmatrix} J_{11} & J_{12} \\ J_{12} & J_{22} \end{pmatrix} \qquad (15)$$

The different elements of the FIM are given by:

$$J_{11}=E\left[\left(\frac{\partial L}{\partial\theta}\right)^2\right]=\xrightarrow[\text{score mean is always } 0]{}=Var\left[\left(\frac{\partial L}{\partial\theta}\right)\right]=$$

$$=Var\left[\frac{\alpha}{\sigma^2}\sum_{n=1}^{N}\frac{\partial g_n(\theta)}{\partial\theta}y_n-\underbrace{\frac{\alpha^2}{\sigma^2}\sum_{n=1}^{N}\frac{\partial g_n(\theta)}{\partial\theta}g_n(\theta)}_{\text{constant per }\theta}\right]=Var\left[\frac{\alpha}{\sigma^2}\sum_{n=1}^{N}\frac{\partial g_n(\theta)}{\partial\theta}y_n\right]= \tag{16}$$

$$\left(\frac{\alpha}{\sigma^2}\right)^2\sum_{n=1}^{N}Var\left[\frac{\partial g_n(\theta)}{\partial\theta}y_n\right];\ (y_n\ \text{are statistically independent})$$

$$=\left(\frac{\alpha}{\sigma^2}\right)^2\sum_{n=1}^{N}\left(\frac{\partial g_n(\theta)}{\partial\theta}\right)^2\sigma^2,\quad \sigma^2=Var[y_n]$$

$$J_{11}=\frac{\alpha^2}{\sigma^2}\sum_{n=1}^{N}\left(\frac{\partial g_n(\theta)}{\partial\theta}\right)^2 \tag{17}$$

$$J_{22}=Var\left[\frac{\partial L}{\partial\alpha}\right]=Var\left[\frac{1}{\sigma^2}\sum_{n=1}^{N}y_n g_n(\theta)\right]=$$
$$\frac{1}{\sigma^4}\sum_{n=1}^{N}g_n^2(\theta)\sigma^2=\frac{1}{\sigma^2}\sum_{n=1}^{N}g_n^2(\theta) \tag{18}$$

$$J_{12}=E\left[\frac{\partial^2 L}{\partial\theta\partial\alpha}\right]=$$
$$E\left[\frac{\partial}{\partial\theta}\left(\frac{1}{\sigma^2}\sum_{n=1}^{N}y_n g_n(\theta)-\frac{\alpha}{\sigma^2}\sum_{n=1}^{N}g_n{}^2(\theta)\right)\right]= \tag{19}$$
$$\frac{1}{\sigma^2}E\left[\sum_{n=1}^{N}y_n\frac{\partial g_n(\theta)}{\partial\theta}-2\alpha\sum_{n=1}^{N}g_n(\theta)\frac{\partial g_n(\theta)}{\partial\theta}\right]$$

Using equation (6) under the assumption that the noise mean is zero:

$$E[y_n]=\alpha g_n(\theta) \tag{20}$$

Substituting equation (20) into equation(19):

$$J_{12}=\frac{1}{\sigma^2}\left(\sum_{n=1}^{N}\alpha g_n(\theta)\frac{\partial g_n(\theta)}{\partial\theta}-2\alpha\sum_{n=1}^{N}g_n(\theta)\frac{\partial g_n(\theta)}{\partial\theta}\right)=$$
$$\frac{-\alpha}{\sigma^2}\left(\sum_{n=1}^{N}g_n(\theta)\frac{\partial g_n(\theta)}{\partial\theta}\right) \tag{21}$$

The CRLB matrix is given by:

$$CRLB(\underline{\theta}) = J^{-1}(\underline{\theta}) = \frac{1}{J_{11}J_{22}}\begin{pmatrix} J_{22} & J_{12} \\ J_{12} & J_{11} \end{pmatrix} \tag{22}$$

The lower bound for the angle estimation error is therefore:

$$CRLB(\theta) = \frac{J_{22}}{J_{11}J_{22} - J_{12}^2} \tag{23}$$

Next, we must calculate the derivative of $g_n(\theta)$ with respect to θ. For the frequency domain case (Gammatone model), this derivative must be estimated numerically, but for the time domain model it can be calculated analytically as follows:

$$\begin{aligned} &\frac{\partial}{\partial\theta}\left(g_n(\theta)\right) = \frac{\partial}{\partial\theta}\left(\sum_{p=1}^{M} x_p[n]A_p[n]h_p[\theta,n]\right) = \\ &\sum_{p=1}^{M} x_p[n]A_p[n]\frac{\partial}{\partial\theta}h_p[\theta,n] \end{aligned} \tag{24}$$

Recall:

$$h_p[\theta,n] = h\left(\theta, f_p(nT)\right) \tag{25}$$

Let f be defined as: $f \triangleq f_p(nT)$, it can be seen that

$$\frac{\partial}{\partial\theta}h_p[\theta,n] = \frac{\partial}{\partial\theta}h(\theta,f)\bigg|_{f=f_p(nT)} \tag{26}$$

So we must calculate the derivative of $h(\theta,f)$.

Observing the piston model equation and marking:

$k \triangleq \frac{2\pi f}{c}$

$\beta \triangleq ka\sin\theta$

We can write 'h' as a function of β:

$$h(\beta)=\frac{2J1(\beta)}{\beta} \tag{27}$$

So the derivative with respect to θ is:

$$\begin{aligned}\frac{\partial h}{\partial \theta}&=\frac{\partial h}{\partial \beta}\frac{\partial \beta}{\partial \theta}\\ \frac{\partial \beta}{\partial \theta}&=ka\cos\theta\end{aligned} \tag{28}$$

$$\frac{\partial h(\beta)}{\partial \beta}=2\frac{J_1'(\beta)\beta-J_1(\beta)}{\beta^2} \tag{29}$$

To calculate $J_1'(\beta)$, we use the first kind Bessel functions derivative rule:

$$\frac{d}{d\beta}\left(\beta^m J_m(\beta)\right)=\beta^m J_{m-1}(\beta) \tag{30}$$

Setting m=1 leads to the following expression for the derivative of the first order Bessel Function:

$$J_1'(\beta)=\frac{d}{d\beta}\left(J_1(\beta)\right)=J_0(\beta)-\frac{1}{\beta}J_1(\beta) \tag{31}$$

Substituting the above expression at equation (29) we obtain:

$$\frac{\partial h(\beta)}{\partial \beta}=2\frac{J_0(\beta)}{\beta}-4\frac{J_1(\beta)}{\beta^2} \tag{32}$$

So the derivative with respect to θ is given by:

$$\begin{aligned}\frac{\partial h}{\partial \theta}&=\left(2\frac{J_0(ka\sin\theta)}{ka\sin\theta}-4\frac{J_1(ka\sin\theta)}{(ka\sin\theta)^2}\right)ka\cos\theta\\ k&\triangleq\frac{2\pi f}{c}\end{aligned} \tag{33}$$

The CRLB is therefore given by equation(23), using equations(24), (25) and (33) to calculate $\frac{\partial}{\partial \theta}\left(g_n(\theta)\right)$ and using equations (17), (18) and (21) to calculate J_{11}, J_{22} and $J_{12.}$ Due to the similarity between the time and frequency domain models, the CRLB calculation is also identical for both cases, except for a derivative calculation that has to be estimated numerically for the Gammatone model case.
